# Crown and Fixed Bridge-Work*Extract from a paper read before the Illinois State Dental Society, Peoria, Illinois, May 10-12, 1921 and printed in *Journal N. D. A.*

**Published:** 1921-11

**Authors:** R. E. MacBoyle


### CROWN AND FIXED BRIDGE-WORK*

BY R. E. MacBOYLE, D.D.S.

The present status of that branch of our profession relating to crown and bridge-work is evidently one of confusion and uncertainty as judged by the various attitudes toward it of many dentists. Many have become radical in their views against fixed bridges, and when a wave of this kind once starts it is generally carried to the extreme.

This branch of dentistry where rightly used is too valuable as a satisfactory means of supplying missing teeth to allow it to remain in its present rather dejected position, and I believe that it is possible to rescue it and bring it into more popular favor.

There is always a cause for every effect and the cause for the confusion and uncertainty regarding fixed bridge-work lies within a chain of conditions which have been thoroughly understood. Stating the case briefly, conditions concerning this work have radically changed within recent years; or, rather, our knowledge of conditions has radically changed, but dentists generally have not changed their methods as required by added knowledge. Dentists have known that something had occurred which demanded different methods, but many have evidently been unable to determine just what it was, and the majority have con-

---

* Extract from a paper read before the Illinois State Dental Society, Peoria, Illinois, May 10-12, 1921 and printed in *Journal N. D. A.*

tinued to employ the old methods not fully realizing the requirements of the new conditions. Many of course did realize that the old methods would not meet their demands successfully, and, not having new and ideal ways to take the place of the old, adopted other means than fixed bridges. This accounts, to a large degree, for the radical wave toward removable appliances, and from this view-point it was justifiable.

The first thing to consider in our analysis is, what brought about the change of knowledge of the conditions concerning fixed bridge-work, and the answer is the X-ray. The next in order is, what has the X-ray taught us, or what should it have taught us, and the answer to this question reveals to us the secret of our confusion and uncertainty.

If we have studied the revelations of the X-ray and interpreted them at all correctly, we have learned, first, that we must conserve the pulps of teeth; second, that we must avoid irritation of the gingival soft tissues; third, that we must procure balanced occlusion with and upon our bridges; and fourth, we must use the proper style of dummies in our fixed bridges, keeping in mind one of the most important qualities necessary, which is cleansableness. If this truly outlines the revelations of the X-ray, then, of course, many of the old methods for many cases are practically obsolete. We must have new ways that are more ideal and more universally adaptable in order to meet the new requirements, and especially where we have sound or practically sound vital teeth to deal with.

Before the advent of the X-ray, we were taught that teeth should be devitalized when used as abutments; that gingival irritation was usually considered as simply a local condition of little importance, and unfortunately it has not been corrected to any great extent. The bad effects of improper occlusion as well as the uncleanliness of improper dummies are conditions which have not as yet impressed themselves seriously upon dentists generally. Devitalization, then, is the only factor which has been corrected to any great extent, and the serious part of this phase of the matter is that, with the cessation of devitalization, we very often find an increase of gingival irritation for the reason that the vital abutment teeth are not prepared as properly for the reception of telescope crowns as were the devitalized ones.

If the foregoing is true, then there is a crying demand for more ideal abutment pieces for vital teeth, and if these can be procured, and if by their use we can avoid the conditions enumerated above, fixed bridge-work will come into its own, the present condemnation of it will cease, and we will, in many cases, supply our patients with missing teeth in the most satisfactory manner. There are other abuses, such as using fixed bridges where spans are too long and where the abutment roots are not sufficiently sound.

A grea deal of extracting both necessary and unnecessary is being done and will continue to be done for some time to come. Thus a demand is created in many cases for the most satisfactory means of supplying one, two, three, and ofttimes, in the anteriors, four missing teeth. The question arises, how are we going to best do this, and I personally believe that the most ideal method in the majority of cases is with a properly constructed and adapted fixed bridge. It simply remains for us to devise ideal abutment pieces and dummies in order to meet the modern demand.

### TELESCOPE CROWNS

As long as we retain broken-down devitalized teeth, there will be a place for the telescope crown. However, it is not suitable for vital posterior teeth for the reason that it causes more or less gingival irritation, due to improperly prepared abutment teeth. We, at least, attempt to teach the proper preparation, but for varied reasons dentists become careless regarding this matter. Just as long as telescope crowns are used upon vital teeth, just so long will they be abused. This is true, no doubt, of all banded crowns, but not to the extent of the telescope.

### PREVENTIVE DENTISTRY

We hear a great deal these days about preventive dentistry. All dentistry for children and adults alike should be preventive from the viewpoint of preventing all irritation. Certainly a great majority of our crowns and fixed bridges have not met this requirement in the past, and if we are to continue this work we must keep in mind the preventive idea as a most important phase of the problem.

### DUMMIES

It has been my observation that ofttimes the dummies used, especially in posterior bridges, are of improper form to best meet the requirements of cleansableness and non-irritation, which are certainly two of the qualities which dummies should possess. The dummies for upper posterior bridges should have no shelves or concavities at the lingual or gingival which make cleansing practically impossible. There should be no saddles and, although the gingival portion of the porcelain should be in contact with the soft tissue, it should not extend too far gingivally, causing it to be in contact with too great a surface. The most universally adaptable dummies at the present time for the upper posteriors are made by using the porcelain pin facing and the metal cusp, for they can be shaped on the lingual with a long sloping surface which the patient can cleanse. These facings should be narrowed at the gingival portion, leaving an interproximal or washable space between them and also between the dummies and the abutments. The porcelain in contact with the soft tissue should be convex in form and highly polished. In long-bite cases the all-porcelain dummies can be used, such as the Goslee for upper posteriors, because they have the long sloping surface at the lingual and allow porcelain in contact with the tissue instead of metal. In the medium or short bites, the porcelain pin facing is best. Regarding the metal cusps in connection with the porcelain pin facing for upper posterior dummies, the metal is not conspicuous if adapted properly. Regarding the width of the cusps from buccal to lingual, the shorter the bite the less this width should be.

For the lower posterior dummies, the gingival should never be in contact with the soft tissue, but a washable space should be left between the dummy and ridge, and in medium or long-bite cases the Goslee tooth is the ideal dummy. In short bites the all metal dummy is necessary, except possibly on the first lower bicuspid, where a porcelain facing should be used in order to avoid the buccal display of metal. The all-porcelain teeth should be used for lower posterior dummies wherever possible for the reason that it is the occlusal surface which is conspicuous in the lower posterior bridges. For the anterior dummies, porcelain of some variety must be used, but the necessary cleansable qualities should not be lost sight of. Whether the porcelain facings of pin or Steele variety or the Goslee be used depends upon the length of bite and general conditions.

### INDICATIONS FOR FIXED BRIDGE-WORK

The indications for fixed bridge-work, as a rule, are in the smaller cases of one, two, or three missing teeth between two abutments, and in the anterior, in the case of the four missing incisors either upper or lower. Ofttimes we may join the smaller bridges by means of solder, making a larger bridge possessing more than two abutments. The abutment roots should be sound, solid, and not affected by pyorrhea. In posterior cases more than three dummies should never be supplied between two abutments, and if the third molar is the distal abutment three dummies are too many for a fixed bridge unless conditions are very favorable. Full upper or full lower one-piece fixed bridges are generally contra-indicated; in these conditions a removable structure is as a rule indicated. If all of the molars are missing on one side, a fixed bridge should not ordinarily be made for the opposite side, but rather a removable structure made to supply both sides. The same rule would apply to the larger anterior spaces if the posteriors were missing on either or both sides.

An ideal abutment piece must possess the following qualities: (1) minimum of tooth mutilation; (2) adaptability to vital teeth; (3) non-irritation to gingival soft tissues; (4) require no anesthetic for preparation; (5) cleansableness; (6) avoidance of splitting of the tooth; (7) capable of successful construction by the average dentist; (8) good aesthetic appearance; (9) successful cementation.

Hart J. Goslee, Chicago, Illinois: If you will look back several years, I think you will agree with me that only a few years ago some of us teachers and specialists in this particular line of work thought we had the subject pretty well in hand, and that there was more or less of a standardization of methods and that the field was covered in a manner which assured successful results to a greater or less extent.

Then came the propaganda against pulpless teeth, and the ruthless sacrificing of so many of them. And then came the propaganda against fixed bridge-work and the assertion that it had no place in dentistry, and that everything must be of a removable character.

The particular requirements in connection with the replacement of missing teeth, as I see them and in view of our present knowledge, are these: *first,* a minimum of tooth mutilation; *second,* the avoidance of gingival irritation. For many years there has been a concerted fight against banding the necks of teeth or placing crowns with bands upon the roots of teeth. There has been a continued and vigorous fight against the use of bands because of the fact that they almost invariably produce gingival irritation to a greater or less degree. While this always was and is still true as applied to single crown work, it is also true of any form of attachment heretofore used as a means of supplying missing teeth, either by fixed or removable bridge-work.

It has been said that there is no place in dentistry for the gold shell crown, and the statement has been made as vigorously as was the statement that there was no place in dentistry for fixed bridge-work. I think there is a place in dentistry for the gold telescope crown. In badly broken down pulpless teeth I believe that we can often best make many of them useful for several years to come by means of the gold telescope crown and I am equally confident that there will always be a place for fixed bridge-work.

